# The Dangerous Telephone Box

**Published:** 1920-07-17

**Authors:** 


### "The Dangerous Telephone Box."

*To the Editor of* The Hospital.

Sir,—With a due appreciation of the officials' side of the telephone question, which you quite justifiably printed in your last week's issue, and admitting also that from their own account of the case to which the boxes are subjected, the authorities appear to be fully alive to a potential danger, yet I cannot admit that I drew an exaggerated picture of the insanitary condition of the average box as we find it to-day. Whatever cleansing is nowadays done, the process should certainly be performed more frequently and more thoroughly. Also the bacteriological defence, in spite of the fact that the names of two well-known men are cited, is not necessarily nowadays either convincing or complete. To take only the specific instances of diphtheria and tubercle is dealing with the matter of disease transmission on a very limited scale. Both these infections are caused by relatively large bacteria, and, unless the infected person actually coughs into the receiver, it is doubtful whether such sputum-carried organisms can be expected to find their way into the moisture remaining on the instrument. To give one instance, we know, after so many recent investigations on influenzal and catarrhal disease transference, that the infection in these conditions is carried in the most minute particles of exhaled moisture. Also there are certainly infections harmful to man, which may find their way to the receiver, but which have no effect "when inoculated into guinea-pigs"—*i.e.*, into lower animals. This experimental inoculation is again not conclusive. The science of bacteriology has progressed not a little during the past few years, and, despite the evidence quoted by your correspondent, I feel sure that in the light of more recent medical experience a complete bacteriological examination of the average public telephone instrument would not nowadays result in such assumptions of complete and absolute safety.

I am, Sir, yours faithfully,

The Writer of the Original Article.

